# The Structural Connectivity Pattern of the Default Mode Network and Its Association with Memory and Anxiety

**DOI:** 10.3389/fnana.2015.00152

**Published:** 2015-11-26

**Authors:** Yan Tao, Bing Liu, Xiaolong Zhang, Jin Li, Wen Qin, Chunshui Yu, Tianzi Jiang

**Affiliations:** ^1^Brainnetome Center, Institute of Automation, Chinese Academy of SciencesBeijing, China; ^2^National Laboratory of Pattern Recognition, Institute of Automation, Chinese Academy of SciencesBeijing, China; ^3^Department of Radiology, Tianjin Medical University General HospitalTianjin, China; ^4^Queensland Brain Institute, The University of QueenslandBrisbane, QLD, Australia; ^5^Key Laboratory for NeuroInformation of Ministry of Education, School of Life Science and Technology, University of Electronic Science and Technology of ChinaChengdu, China

**Keywords:** DTI, default mode network, memory, anxiety, structural network

## Abstract

The default mode network (DMN) is one of the most widely studied resting state functional networks. The structural basis for the DMN is of particular interest and has been studied by several researchers using diffusion tensor imaging (DTI). Most of these previous studies focused on a few regions or white matter tracts of the DMN so that the global structural connectivity pattern and network properties of the DMN remain unclear. Moreover, evidences indicate that the DMN is involved in both memory and emotion, but how the DMN regulates memory and anxiety from the perspective of the whole DMN structural network remains unknown. We used multimodal neuroimaging methods to investigate the structural connectivity pattern of the DMN and the association of its network properties with memory and anxiety in 205 young healthy subjects with age ranging from 18 to 29 years old. The Group ICA method was used to extract the DMN component from functional magnetic resonance imaging (fMRI) data and a probabilistic fiber tractography technique based on DTI data was applied to construct the global structural connectivity pattern of the DMN. Then we used the graph theory method to analyze the DMN structural network and found that memory quotient (MQ) score was significantly positively correlated with the global and local efficiency of the DMN whereas anxiety was found to be negatively correlated with the efficiency. The strong structural connectivity between multiple brain regions within DMN may reflect that the DMN has certain structural basis. Meanwhile, the results we found that the network efficiency of the DMN were related to memory and anxiety measures, indicated that the DMN may play a role in the memory and anxiety.

## Introduction

Analyzing task-free functional connectivity using temporal correlations has identified several resting-state networks (RSNs). One of these is the default mode network (DMN; Raichle et al., 2001; Raichle and Snyder, 2007), which has been studied extensively and is widely known to be involved in many brain functions, such as self-referential mental activity and introspective thought processing (Gusnard and Raichle, 2001; Salomon et al., 2009, 2014; Callard and Margulies, 2014). The DMN is a specific brain system that shows greater activity during the resting state than during active cognition (Buckner et al., 2008) and comprises several regions, including the medial prefrontal cortex (mPFC), posterior cingulate cortex (PCC), and medial temporal lobes (MTL; Supekar et al., 2010). Even though previous studies have primarily focused on the function of the DMN using functional neuroimaging techniques, the structural basis of the DMN remains a topic of particular interest.

Several studies have attempted to examine the structural basis of the DMN. The DMN undergoes changes in both functional and structural connectivity during development, as shown by a study that compared children to young adults and revealed that the fiber densities and mean fractional anisotropy (FA) values of the PCC-mPFC tracts and the PCC-left MTL tracts were all significantly lower in the children (Supekar et al., 2010). The mPFC-PCC connectivity in the DMN was found to be immature in children, and a weak (not significant) positive correlation was demonstrated between the mPFC-PCC connectivity and the FA value of the cingulum tract, in contrast to adults, who showed a significant positive relationship (van den Heuvel et al., 2008; Supekar et al., 2010; Gordon et al., 2011). When studying the PCC/retrosplenial cortex (RSC) as the region of interest (ROI) using DTI fiber tractography, the mPFC was found to connect to the more rostral aspect(PCC) of the ROI, whereas the MTLs connected to the more caudal aspect (RSC; Greicius et al., 2009). Most of the previous studies have focused on a few regions or white matter tracts of the DMN so that the global structural connectivity pattern and network properties of the DMN remain unclear.

The brain regions of the DMN are associated with a variety of brain functions (Broyd et al., 2009). Evidences have indicated that the DMN may be involved in regulating memory performance and anxiety levels. In particular, the PCC is concerned with memory and self-referential processing (Kjaer et al., 2002); the medial temporal lobe, including the hippocampus (HIP), is engaged in declarative memory (including episodic memory; Milner, 2005; Viard et al., 2007); the medial frontal lobe is activated during theory of mind processes (Buckner and Carroll, 2007); the mPFC is related to the integration of cognition and emotional processing and is involved with anxiety (Gusnard et al., 2001; Zhao et al., 2007); and semantic memory processing and spatial attention are related to the inferior parietal cortex, specifically the angular gyrus (Binder et al., 2009). Moreover, the DMN has been reported to be disrupted in various memory-related or anxiety-related disorders, such as depression, bipolar disorder (BD), and Alzheimer's disease (AD). Impaired connectivity between the PCC and the HIP and changes in activity within the PCC and the HIP were found in AD, and these effects may be regarded as early markers for AD (Zhou et al., 2010; Mevel et al., 2011). The main differences between the DMN activity in BD patients and healthy controls were found in the connectivity of the mPFC and the anterior cingulate cortex (ACC) with limbic-striatal structures in the DMN (Vargas et al., 2013). Increased activity in DMN regions such as the subgenual cingulate was found in patients with major depression, and the functional connectivity in the subgenual cingulate was positively correlated with the length of the current depressive episode (Greicius et al., 2007; Sheline et al., 2009).

Network analyses of the brain using graph theory can provide information about the integration and segregation of the brain networks. However, very little research has focused on the DMN structural network, and how the DMN regulates memory and anxiety from the perspective of the whole structural network of the DMN remains unknown.

The goals of the present study were (1) to investigate the structural connectivity pattern of the DMN using multimodal neuroimaging methods and (2) to delineate the association of the DMN network properties with memory and anxiety in young healthy subjects. Using an independent component analysis (ICA) method based on resting-state functional MRI, we identified the DMN in this population and selected eight brain regions as seeds. Then, to obtain a picture of the structural connectivity pattern of the DMN, we used a probabilistic tractography technique to track the white matter fibers within the DMN and obtain the probability of fibers connecting any two regions. We used graph theory with the DMN network to compute the network properties and to analyze whether behavioral characteristics, especially memory and anxiety, are associated with the network properties of the DMN.

## Material and methods

### Subjects

A total of 205 Han Chinese healthy adults (80 males) participated in this study and underwent functional magnetic resonance imaging (fMRI), DTI, and T1 scans. The ages of the participants ranged from 18 to 29 years [mean age: 22.88 years (SD: 2.32 years)]. All the subjects were carefully questioned to ensure that they had no family history of psychiatric disorders, drug or alcohol abuse, psychiatric or neurological illness, head trauma, or contraindications to receiving an MRI scan. The study was approved by the local Medical Research Ethics Committee of the Tianjin Medical University. All the subjects signed written informed consent forms.

All the subjects were examined using the Chinese Revised Wechsler Adult Intelligence (WAIS-RC; Gong, 1982) and Memory Scale (WMS-RC) (Gong et al., 1989). The Self-Rating Anxiety Scale (SAS) (Zung, 1971) was used to measure anxiety for each subject. The full-scale intelligence quotient (IQ) ranged from 85 to 139 [mean IQ: 118.09 (SD: 8.45)]. The Memory Quotient (MQ) ranged from 92 to 140 [mean MQ: 116.36 (SD: 9.03)], and the SAS score ranged from 20 to 46 [mean SAS: 28.97 (SD: 4.87)].

### MRI data acquisition

All scans were conducted using the same magnetic resonance scanner (SignaHDx 3.0T, GE Healthcare; Milwaukee, WI, USA).

The high-resolution T1 weighted brain volume was collected with a 3D MRI sequence [repetition time (TR) = 8.1 ms; echo time (TE) = 3.1 ms; voxel size = 1 × 1 × 1 mm^3^; flip angle = 13°; 176 slices].

Resting-state fMRI data was obtained with a single-shot gradient echo-planar imaging sequence (TR = 2 s; TE = 30 ms; matrix size = 64 × 64; field of view (FOV) = 240 × 240 mm^2^; voxel size = 3.75 × 3.75 × 4.0 mm^3^; 40 slices; 180 volumes; flip angle = 90°). All subjects were required to close their eyes and remain still without falling asleep.

DTI data was obtained using an echo planar imaging sequence [TR = 1 s; TE = 64 ms; matrix size = 128 × 128; field of view = 256 × 256 mm^2^; voxel size = 2.0 × 2.0 × 3.0 mm^3^; flip angle = 90°; b0 = 0 (3 repeated acquisitions), b = 1000 s/mm^2^, 55 directions; slice thickness = 3 mm, 45 slices].

During the scanning process, we took measures, such as a foam pad and ear plugs for each participant, to reduce head motion and noise.

### Data preprocessing

The preprocessing of the fMRI data was carried out using the brainnetome fMRI toolkit(BRAT) which is an in-house software in our lab, and is used to conduct the preprocessing steps of the fMRI data and the construction and analysis of the network (http://www.brainnetome.org/en/brainnetometool.html) and included the following steps: (1) discarding the first 10 volumes; (2) slice timing correlation; (3) correcting for head motion (realigning all of the volumes to the first volume);and (4) spatial normalization to a standard space using the EPI template in MNI.

The preprocessing of the DTI data included the following steps: (1) DICOM data conversion; (2) extracting a brain mask; (3) correcting for eddy current and head motion; (4) fitting diffusion tensors to the data. All data preprocessing and processing procedures for the DTI data were implemented in the PANDA software, which is a matlab toolbox for pipeline processing of DTI images and the integrated processing modules of some established packages, including FSL, Diffusion Toolkit and so on, were employed in it(http://www.nitrc.org/projects/panda/) (Cui et al., 2013).

### Definition of the DMN and construction of structural connectivity

**Figure 1:**
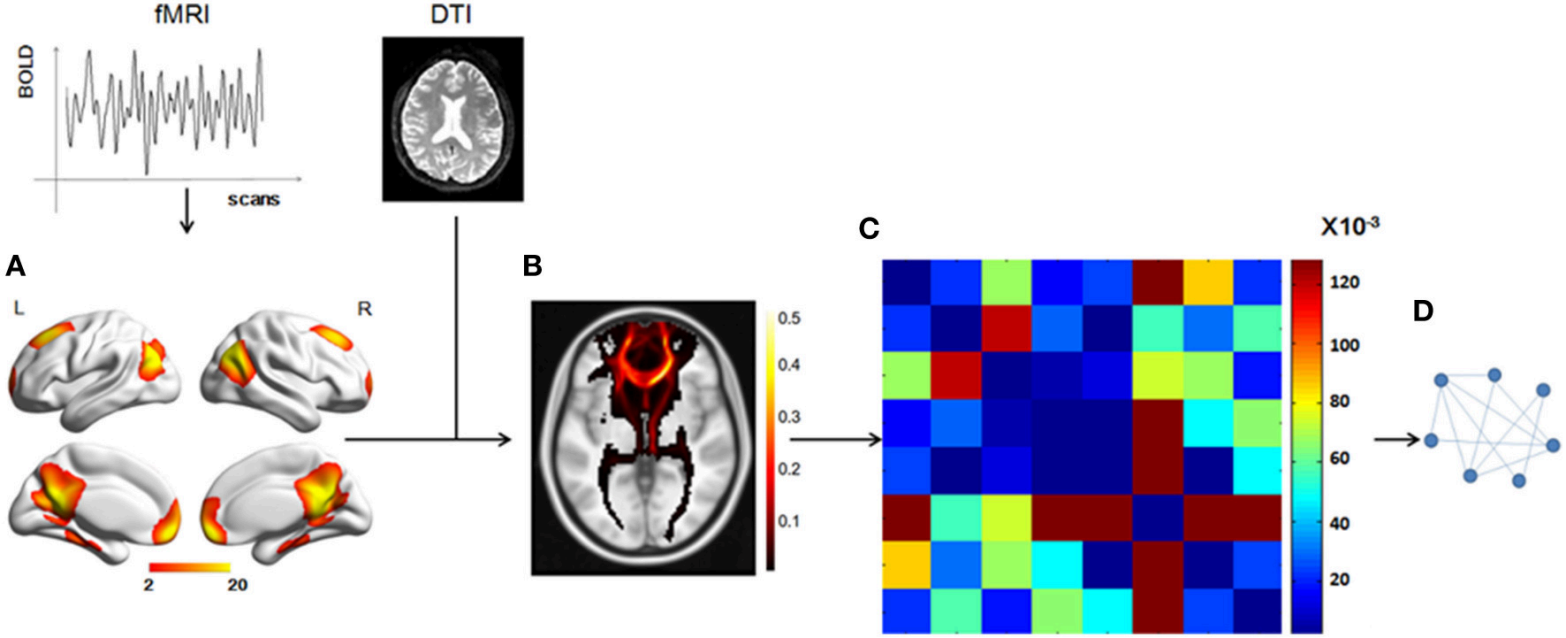
**Total workflow before the statistical analysis. (A)** The DMN mask was extracted from fMRI data using group ICA, with a threshold of *z* = 2. **(B)** A probabilistic fiber tractography method was used to track the fibers from each ROI and the connectivity probability between any two ROIs was calculated to get the matrix **(C)**. **(D)** A graph theory method, in which the nodes represented the ROIs and the weighted edges reflected the connectivity probabilities between any two nodes, was used to analyze the DMN structural network.

The workflow before the statistical analysis is shown in Figure 1. All the steps were carried out to obtain a structural network of the DMN.

We used a data-driven method to extract the mask of the DMN, using the Group ICA from the fMRI Toolbox (GIFT; http://mialab.mrn.org/software/gift/index.html) for the group ICA analysis. We set the number of independent components to be 20. To identify the DMN component among the 20 components, the group components were correlated with the DMN template in the GIFT toolbox and the most relevant component was chosen. Then, in this component map, we set a threshold of z > 2 to obtain a DMN mask of eight ROIs [the mPFC, the PCC, the left and right superior prefrontal cortices (supF), the left and right HIP, and the left and right temporoparietal regions (TP)] and define the DMN.

We chose a probabilistic tractography method to track the white matter fibers and construct the DMN structural network. The fiber tracking for each individual was conducted in native DTI space and included two steps. First, for each voxel, the probability distribution of the primary diffusion direction was estimated using Markov Chain Monte Carlo (MCMC) sampling methods with Bedpostx. Second, the distribution of white matter connections between the seed and target regions was estimate using Probtrackx. The seed regions were the eight ROIs, which were transformed into individual DTI space using FNIRT. Within each seed ROI, 5000 streamline samples were initiated from all voxels and tracked along the probability distribution of the primary diffusion direction of the local voxels until they terminated in voxels within other seed regions. The step length and curvature threshold were set by default values (0.5 mm, 0.2, respectively). The probability of structural connectivity from region i to region j was defined as the number of fibers passing through region j from region i divided by the number of fibers that were sampled in region i (5000 × number of voxels in region i). We applied the track tracing in both directions and the probability of structural connectivity between region i and region j was computed as the average of the probability from region i to region j and from region j to region i.

**Figure 2:**
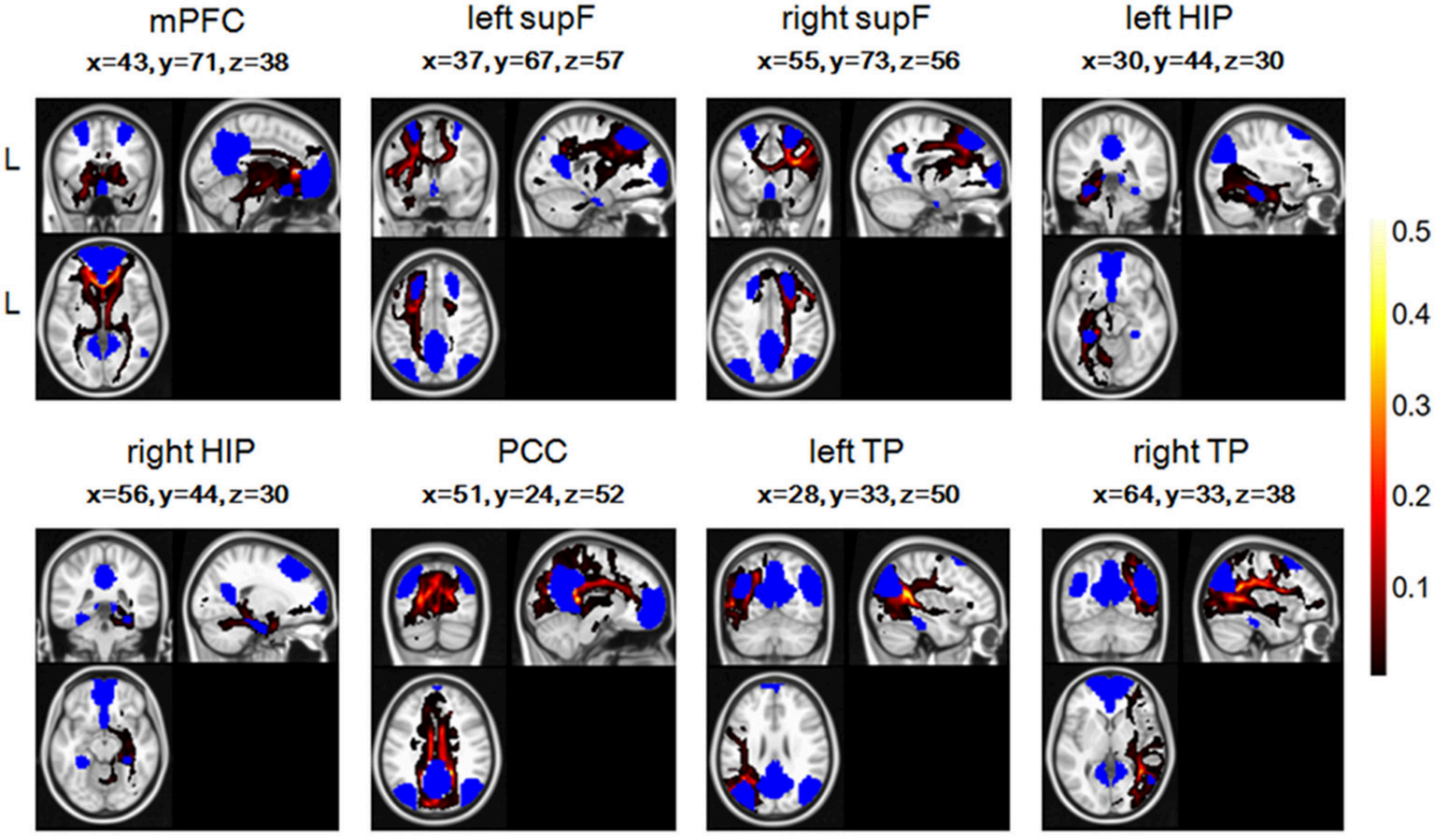
**The fiber tracts of the probabilistic tractography of eight regions in the DMN and their coordinates in one single subject, with blue representing the eight seed regions**. The color of each voxel represented the number of fibers passing through this voxel divided by the total number of fibers sampled from the eight seed regions.

To combine or compare the results from all the subjects, we transferred the fiber tracts from the native space to the standard space using the FMRIB Software Library (FSL; http://fsl.fmrib.ox.ac.uk/fsl/fslwiki/FSL) with the following steps. First, the T1 image was coregistered to the b0 image (the volume with no diffusion weighting) using an affine transformation (FLIRT in FSL) and naming the output image as rT1. Second, the rT1 image was normalized into the MNI space using a nonlinear transformation (FLIRT and FNIRT in FSL), and the fiber tracts in DTI space were transferred into standard space using the same nonlinear transformation matrix. The fiber tracking images of a single subject in standard space were shown in Figure 2, in which the color of each voxel represented the number of fibers passing through this voxel divided by the total number of fibers sampled from the eight seed regions.

Based on the structural connectivity of the DMN, we used the mean structural connectivity to measure the power of the connectivity, which is defined as ${\bar{A}}_{ij}=\frac{1}{num}\underset{subject}{\sum }\;A_{ij}$ (num is the number of subjects and *A*_*ij*_ is the probability of connection between region i and region j).

### Network properties

The results of the fiber tracking described above included a matrix in which any element *A*_*ij*_ represented the probability of structural connectivity between region i and region j. We then described the matrix as a weighted graph for each individual in which the node represented the regions in the DMN and the edge represented the probability connecting two regions in that matrix. In the present study, graph theory was used to study the network (Achard and Bullmore, 2007; Li et al., 2009), and the network properties were computed using in-house code.

#### Average degree

The degree of every node *D*_*i, i* = 1, 2, 3, …*N*_ is defined as the sum of all the structural connectivity connecting to it from other nodes. The average degree of a network is the average of the degree of all nodes:

$$\begin{array}{l} D=\frac{1}{N}\overset{N}{\underset{i=1}{\sum }}D_{i} \end{array}$$

#### Clustering coefficient

In a weighted network, the clustering coefficient for each node is defined as:

$$\begin{array}{l} C_{i}=\frac{1}{S_{i}\left(D_{i}\;-\;1\right)}\underset{j,h}{\sum }\frac{w_{ij}\;+\;w_{ih}}{2}a_{ij}a_{ih}a_{jh} \end{array}$$

*a*_*ij*_ equals 1, if node i is connected to node j and otherwise equals 0. w_ij_ is defined as the strength of the connectivity between node i and node j. $S_{i}=\underset{j}{\sum }a_{ij}w_{ij}$ is defined as the sum of the strength of the connectivity that connects to node i from all other nodes. The average clustering coefficient of the network is measured using the following equation:

$$\begin{array}{l} C=\frac{1}{N}\underset{i\in G}{\sum }C_{i} \end{array}$$

which measures the local structure of the network.

#### Shortest path length

The shortest path length of node i is defined as

$$\begin{array}{l} L_{i}=\frac{1}{N-1}\underset{\begin{array}{c} i,j\in G\\ i\neq j \end{array}}{\sum }d_{ij} \end{array}$$

where d_ij_ represents the shortest path length between node i and node j. We can use algorithms, such as that of Dijkstra (1959), to calculate this length. The average shortest path length of the network is defined as:

$$\begin{array}{l} L=\frac{1}{N}\underset{i\in G}{\sum }L_{i} \end{array}$$

which measures the extent of the average connectivity and reflects the routing efficiency in the graph.

#### Global efficiency

The global efficiency of the network is defined as:

$$\begin{array}{l} E_{global}=\frac{1}{N\left(N-1\right)}\underset{\begin{array}{c} i,j\in G\\ i\neq j \end{array}}{\sum }\frac{1}{d_{ij}} \end{array}$$

which reflects the ability of parallel information transfer in the network.

#### Local efficiency

The local efficiency of node i is defined as *E*_*i*_ = *E*_*global*_(*G*_*i*_), where *G*_*i*_ is composed of the nodes that connect to node i directly (not including node i) and the interconnected edges. The local efficiency measures the fault tolerance of the network and indicates how well the information is exchanged in the subgraph. The average efficiency is measured using the following equation:

$$\begin{array}{l} E_{local}=\underset{i\in G}{\sum }E_{i} \end{array}$$

#### Nodal efficiency

The efficiency of node i is defined as:

$$\begin{array}{l} E_{nodal\left(i\right)}=\frac{1}{N-1}\underset{\begin{array}{c} i,j\in G\\ i\neq j \end{array}}{\sum }\frac{1}{d_{ij}} \end{array}$$

This measure quantifies the significance of each node for the communication in the network and nodes with high nodal efficiencies can be regarded as hub nodes (Achard and Bullmore, 2007; Gong et al., 2009; Wang et al., 2012).

### Statistical analysis

The behavioral characteristics analyzed in this study were the MQ and the SAS score. We used partial correlation coefficients between the MQ, the SAS score, and the DMN network properties to study the relationship between the DMN and memory and the relationship between the DMN and anxiety. The partial correlation was computed by factoring out the effects of age, gender, and IQ [the MQ was significantly positively correlated with the IQ (*p* < 0.01)]. We also analyzed the relationship between the MQ, SAS score and the structural connectivity and the relationship between the MQ, SAS score and the nodal properties, such as nodal degrees and nodal efficiencies. Since previous studies have demonstrated the effect of brain size on anatomical network organization (Yan et al., 2011), we also controlled the brain size in the analysis, which was calculated with T1 data using voxel-based morphometry (VBM) method in SPM8 software (http://www.fil.ion.ucl.ac.uk/spm/).

## Results

### Structural connectivity pattern of the DMN

**Figure 3:**
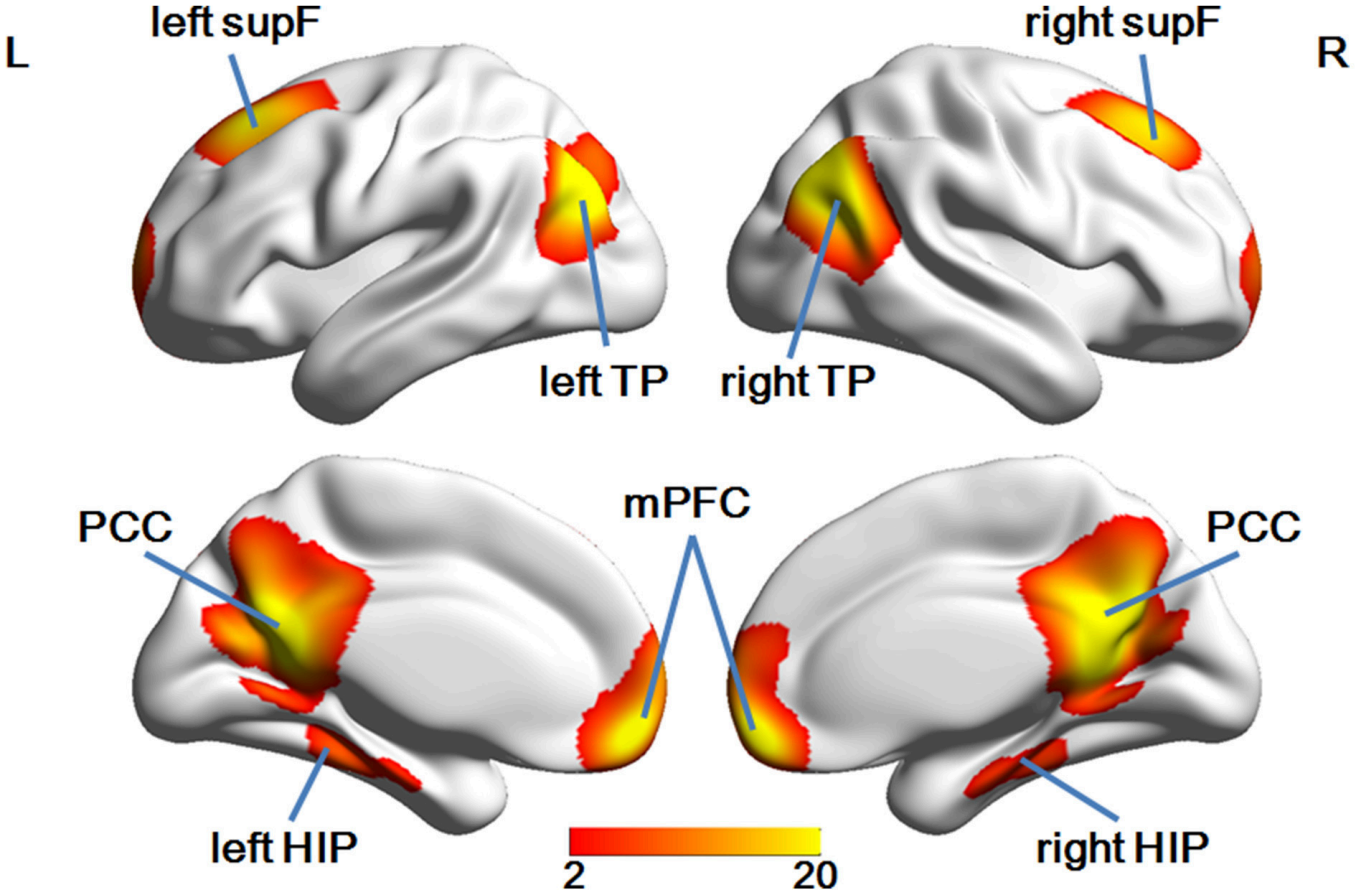
**The eight ROIs identified in the DMN mask**.

Group ICA analysis identified eight ROIs in the DMN mask (Figure 3), a finding which was in good accordance with a few other studies (Liu et al., 2012; Pluta et al., 2014).

**Figure 4:**
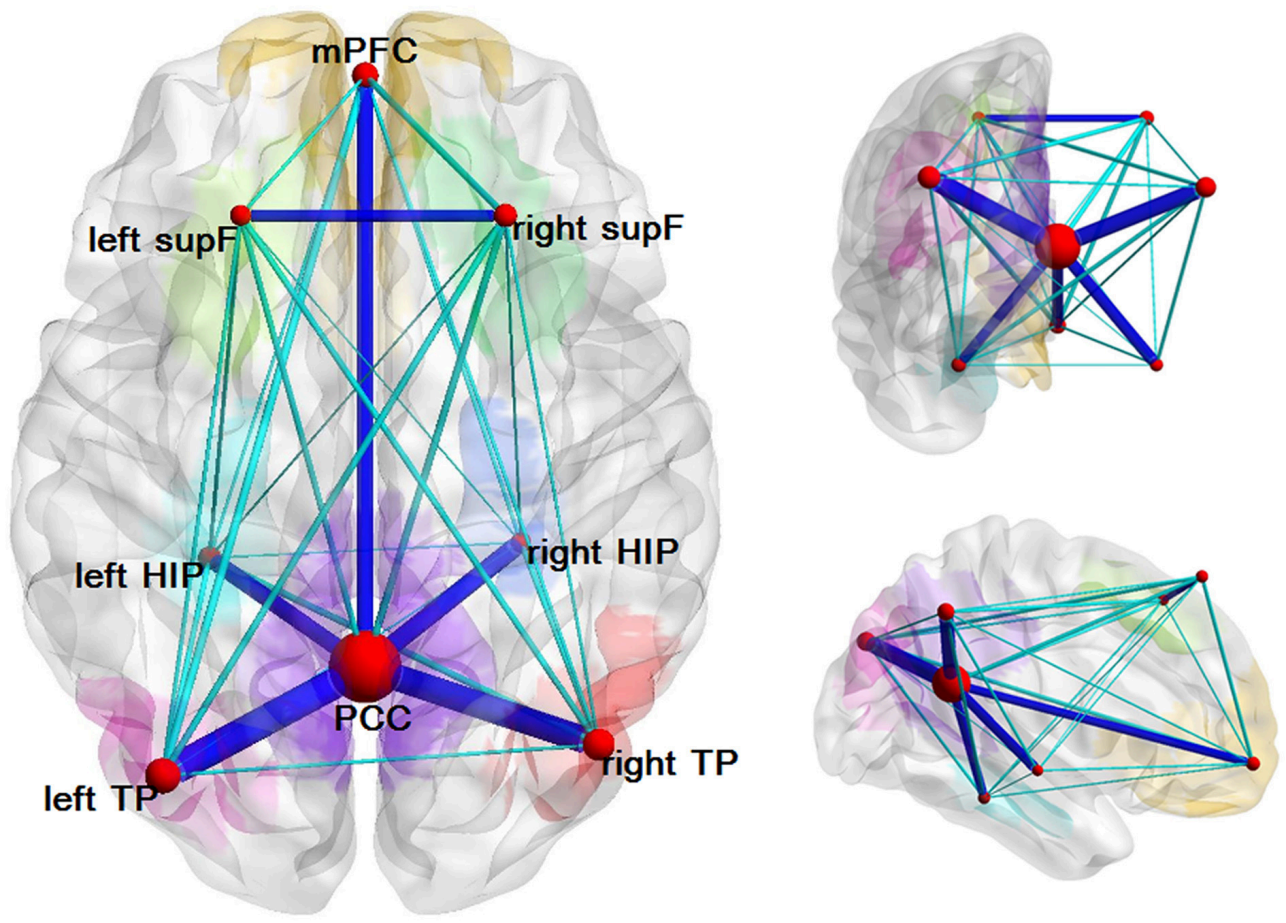
**Structural network of the DMN**. Eight ROIs and the structural connectivity between any two regions are shown in this graph. The size of the node corresponds to the degree of the node and the connectivity was thresholded by 0.1 with dark blue indicating values greater than 0.1 and light blue indicating values less than 0.1.

We found several structural connectivities, such as the connectivities between the PCC and the left TP, the PCC and the right TP, the right HIP and the PCC, the left HIP and the PCC, the mPFC and the PCC, the left supF and the right supF, that were relatively higher than the rest. As is clear from this list, most of these connectivities were related to the PCC. In addition, some structural connectivities, such as the connections between the left supF and the right HIP, the right HIP and the left TP, and the left HIP and the right HIP (Figure 4), were found to be relatively small.

### Relationship between memory, anxiety, and the structural network properties of the DMN

First, we examined the relationship between the MQ and the SAS score and the correlation was significant (*r* = −0.223, *p* = 0.001). Then we examined the partial correlations between the MQ, the SAS score, and network properties. Before analyzing the relationship, we applied a threshold to remove those connections with small probabilities in the network. Since there is no definitive way to select the optimal threshold, we chose a range of thresholds between 0 and 0.01 with intervals of 0.0005(in total of 20 thresholds). Only the results under thresholds from 0.001 to 0.005 were shown in Table 1, because when the threshold ≥ 0.006, the network of some subjects became disconnected. The results showed that the MQ was significantly positively correlated with D, Eglobal, and Elocal, while these network properties were significantly negatively correlated with the SAS score all the time under different thresholds (Table 1). Besides, the correlation between the clustering coefficient and SAS score became significant when the threshold was greater than 0.001. We did not find significant correlations between the shortest path length and the MQ, SAS score under the thresholds ranging from 0 to 0.005.

**Table 1:** **Partial correlations between the MQ, SAS scores, and the global network properties of the whole DMN with or without controlling the brain size under different thresholds**.

**Threshold**						**Controlling brain size**			
		**MQ**		**SAS**		**MQ**		**SAS**	
		***pc***	***p-value***	***pc***	***p-value***	***pc***	***p-value***	***pc***	***p-value***
0.001	D	0.130	0.034^*^	−0.164	0.010^*^	0.133	0.032^*^	−0.167	0.009^*^
	C	0.076	0.143	−0.073	0.151	0.079	0.134	−0.076	0.141
	L	−0.067	0.175	0.083	0.120	−0.070	0.163	0.087	0.111
	E_global_	0.145	0.021^*^	−0.147	0.019^*^	0.148	0.019^*^	−0.150	0.017^*^
	E_local_	0.151	0.017^*^	−0.155	0.014^*^	0.152	0.016^*^	−0.156	0.013^*^
0.002	D	0.130	0.034^*^	−0.164	0.010^*^	0.133	0.032^*^	−0.167	0.009^*^
	C	−0.007	0.462	−0.128	0.035^*^	0.000	0.499	−0.137	0.027^*^
	L	−0.067	0.175	0.083	0.120	−0.070	0.163	0.087	0.111
	E_global_	0.145	0.021^*^	−0.147	0.019^*^	0.148	0.019^*^	−0.150	0.017^*^
	E_local_	0.138	0.027^*^	−0.168	0.009^*^	0.140	0.025^*^	−0.171	0.008^*^
0.003	D	0.130	0.034^*^	−0.164	0.010^*^	0.132	0.032^*^	−0.167	0.009^*^
	C	−0.019	0.393	−0.207	0.002^*^	−0.012	0.431	−0.217	0.001^*^
	L	−0.067	0.175	0.083	0.120	−0.070	0.163	0.087	0.111
	E_global_	0.145	0.021^*^	−0.147	0.019^*^	0.148	0.019^*^	−0.150	0.017^*^
	E_local_	0.155	0.015^*^	−0.176	0.006^*^	0.156	0.014^*^	−0.177	0.006^*^
0.004	D	0.129	0.035^*^	−0.164	0.010^*^	0.132	0.032^*^	−0.167	0.009^*^
	C	0.031	0.331	−0.150	0.017^*^	0.040	0.286	−0.162	0.011^*^
	L	−0.067	0.175	0.083	0.120	−0.070	0.163	0.087	0.111
	E_global_	0.145	0.021^*^	−0.147	0.019^*^	0.148	0.019^*^	−0.150	0.017^*^
	E_local_	0.176	0.007^*^	−0.171	0.008^*^	0.176	0.007^*^	−0.171	0.008^*^
0.005	D	0.130	0.034^*^	−0.164	0.010^*^	0.133	0.032^*^	−0.167	0.009^*^
	C	0.031	0.333	−0.158	0.012^*^	0.041	0.284	−0.172	0.007^*^
	L	−0.067	0.175	0.083	0.120	−0.070	0.163	0.087	0.111
	E_global_	0.145	0.021^*^	−0.147	0.019^*^	0.148	0.019^*^	−0.150	0.017^*^
	E_local_	0.177	0.006^*^	−0.199	0.002^*^	0.178	0.006^*^	−0.200	0.002^*^

*pc refers to partial correlation coefficient*,

* *means the correlation is significant (p < 0.05)*.

Besides, we controlled the brain size in the statistical analysis of network properties to remove its effect and repeated the analyses above and the significant results remain unchanged, shown in Table 1.

In addition, we analyzed the relationship between the MQ, the SAS score, and the structural connectivity, but there were not any significant association after a correction for multiple comparisons using the false-discovery rate (FDR), *q* < 0.05 correction. The results under the threshold of 0.002 were shown in Supplementary Tables 1, 2.

### Properties of each node and hub roles in the DMN

We analyzed the degree of each node and the nodal efficiencies. As high nodal efficiency implies hub roles in the network and can be regarded as hub nodes (Achard and Bullmore, 2007; Gong et al., 2009; Wang et al., 2012), we calculated the mean nodal efficiency of each region at each threshold and the results were the same that the PCC had the hightest mean nodal efficiency, followed by the left and right TP and mPFC. The results of the nodal degree were the same as the nodal efficiency, which could also be seen from Figure 4 in which the size of nodes represented the average degree of the nodes.

Furthermore, the degrees of several ROIs, such as the mPFC and PCC, were found to be positively correlated with the MQ, but none of the correlations was significant after FDR, *q* < 0.05 correction. Only the correlation between the degree of the mPFC and the SAS score remained significant, although the degrees of most of the ROIs were negatively correlated with the SAS score. The efficiency of mPFC was also significantly negatively correlated with the SAS score and the efficiency of three regions exhibited significant association with the MQ, including mPFC, PCC, and right TP after FDR, *q* < 0.05 correction. Since the results were similar, we only displayed the results under the threshold of 0.002 in Supplementary Tables 1, 2.

## Discussion

We used a multimodal analysis, combining fMRI with DTI, to analyze the structural connectivity pattern of the DMN and the regulating effects of network properties on behaviors in the DMN. We found that the correlations between the MQ and D, E_global_, and E_local_ were always significantly positive, whereas the correlations between D, E_global_, E_local_, and the SAS score were always significantly negative under different thresholds. The connectivity probability in our study was computed as the probability between any two regions in the DMN which was usually used in previous studies(Gong et al., 2009; Cao et al., 2013; Li et al., 2013). We also included the mean FA along the connection when calculating the connection strength and reanalyzed the associations with memory and anxiety. The general results did not change and the associations were still significant or marginally significant. Besides, as previous studies demonstrated that the size of brain may affect the anatomical network organization, we also controlled the brain size in the statistical analysis of the network properties to remove its effect, but the significant results remain unchanged, which indicated that the brain size may not change the overall characteristics of the network in the brain. In our study, we did not find significantly correlations between the structural connectivity and MQ, SAS, while some other studies reported the relationship between the anatomical connectivity and cognitive score, such as memory (Andrews-Hanna et al., 2007; Vidal-Piñeiro et al., 2014). One reason for this is that most of these studies examined the old people while our study focused on young health subjects, which in turn suggested that the network attributes may be more sensitive to the differences of cognition and emotions than the structural connectivity. These findings help to validate the theory that, when it is regarded from a network perspective, the DMN can be seen to play a significant role in memory and anxiety.

In this study, we tracked the white matter tracts in the DMN and found several structural connectivities, such as the PCC-the left TP, the PCC-the right TP, the right HIP-the PCC, the mPFC-the PCC, the average connection probabilities of which were relatively higher than others, which indicated that the DMN has definite structural basis, as can be seen in Figure 4. Most of the regions with relatively high structural connectivities were related to the PCC or the mPFC, and some of which have also been reported or examined by other studies, such as HIP-PCC (Teipel et al., 2010) and the mPFC-PCC (van den Heuvel et al., 2008; Greicius et al., 2009; Supekar et al., 2010; Gordon et al., 2011). However, we may keep cautious to explain several relatively low structural connectivity, such as the left supF-the right HIP, the right HIP-the left TP, the left HIP-the right HIP. To date, a few studies have begun to elucidate the relationship between structural connectivity and functional connectivity in the brain using multimodal analysis on data from fMRI and DTI. With analyses that inferred functional connectomes from structural connectomes (excluding indirect structural connections) and that inferred structural properties from functional properties, a study by Hermundstad et al. (2013) described a robust relationship between structural connectivity and functional connectivity. Some other studies have found that functional connectivity can exist even without direct structural connectivity (Greicius et al., 2009; Honey et al., 2009; Tyszka et al., 2011).

The DMN is an important brain network in terms of self-referential mental activity and introspective thought processing. The DMN also seems to be engaged in memory and emotion (including anxiety). The left HIP, mPFC, left temporal lobe, bilateral temporoparietal junctions, and PCC have been found to be activated by certain types of memories (Maguire and Mummery, 1999; Maddock et al., 2001). The ventral HIP and mPFC were found to play a significant role in anxiety (Kjelstrup et al., 2002; Vertes, 2004; Adhikari et al., 2010). We focused our attention on the relationships between memory, anxiety, and the properties of the DMN from a network perspective using graph theory. Graph metrics, such as the average degree, clustering coefficient, shortest path length, global efficiency, and local efficiency, can accurately describe a graph or network. The clustering coefficient measures the extent of local cliquishness of the network and the mean shortest path length is a measure of the integration of the network by estimating the effective communication between brain regions. The local efficiency reflects the fault tolerance of the network, which indicates the ability of each subgraph exchanging information if the current node is eliminated and the global efficiency may reflect the features of the brain, such as the neurotransmitter release at synapse and the propagation of action potential along axons(Latora and Marchiori, 2001; Achard and Bullmore, 2007; Rubinov and Sporns, 2010; Li et al., 2013). Our study was the first to examine the relationship between the DMN and memory, anxiety from the structural network point of view and the global and local efficiency were found to be significantly correlated with the MQ and SAS score, indicating that a higher MQ corresponded to higher global and local efficiencies of the DMN network, while a higher level of anxiety corresponded to lower global and local efficiencies. Several previous studies have reported the reduction of efficiency in the network in diseases with memory impairment or depression, such as schizophrenia, BD (Wang et al., 2010; Kim et al., 2013; Ajilore et al., 2014). In the analysis of the nodal properties, several regions with hub roles in the DMN were found, including PCC, left and right TP, and mPFC. In addition, memory may be more related to the PCC, mPFC, and right TP, while anxiety related to mPFC, with the analysis of the relationship between the average degree of node, nodal efficiency and memory, anxiety, shown in Supplementary Tables 1, 2, which have also been reported in other studies(Vogt et al., 1992; Drevets, 2001; Nielsen et al., 2005; Zhao et al., 2007; Euston et al., 2012). The PCC has been found to be related to certain types of memory retrieval, cognitive control, and integration (Maguire and Mummery, 1999; Maddock et al., 2001; Buckner et al., 2008; Leech et al., 2012), and the mPFC has been found to be related to functions of the theory of mind and the integration of cognition and emotion, as well as to personal, time-specific memories (Maguire and Mummery, 1999; Gusnard et al., 2001; Buckner et al., 2008). Abnormalities in the PCC and the mPFC may be associated with certain diseases and impairments in brain function. For example, increased functional connectivity in the PCC and damages in the cingulum tract were observed to be correlated with working memory performance and attention in schizophrenia (Kubicki et al., 2003; Whitfield-Gabrieli et al., 2009); reduced PCC deactivation during a task was related to the impaired social functions associated with autism spectrum disorder (Kennedy et al., 2006). In addition, disrupted connections between the PCC and mPFC have been implicated in impaired cognitive functions, including the process of working memory, in schizophrenia and BD (Chai et al., 2011; Whitfield-Gabrieli and Ford, 2012), and abnormalities in the mPFC have been suggested to be linked to impaired emotion regulation in BD (Phillips et al., 2008). The results in our study add to the evidence for the functions of the PCC and the mPFC.

Recently, several studies started to examine the relationship between anxiety and memory and found a significant association between the memory impairment and anxiety symptom (Marzi et al., 2014; Crespo et al., 2015). In our study, significant negative correlation was found between memory and anxiety (*p* = 0.001) and the significant correlations with the DMN properties were also found, which will help to add evidence for the relationship among memory, anxiety, and the DMN.

Previous studies have indicated the structural connectivity of the DMN may be disrupted in the populations with anxiety disorders or memory impairments. For example, in Mild Cognitive Impairment (MCI) group, structural disconnection in the DMN was observed especially in the links between inferior parietal and posterior cingulate compared to controls (Garcés et al., 2014). Some other white matter connectivities of DMN have also been reported to be disrupted in AD and MCI, such as cingulum, parahippocampal bundle (Bozoki et al., 2012; Weiler et al., 2014). However, if the associations between DMN and memory or anxiety still exist in populations with anxiety disorders or memory problem keeps unclear and needs further study.

There were several limitations of this present study. First, the sample of our study was young healthy Chinese subjects. More studies from other independent populations are needed to replicate the current findings. Second, the DMN were reported to be engaged in emotion regulation and this study reported the relationship between the DMN and anxiety level. However, other major emotional problems, e.g., depression, may also be greatly related to the DMN (Coutinho et al., 2015; Shi et al., 2015). More comprehensive emotional measures should be considered in the future studies.

Overall, although the subjects in our study were healthy adults, our findings suggest that the network properties of the DMN or the properties of certain ROIs may change in memory- or anxiety-related diseases. Future studies should be aimed at examining the DMN in certain diseases, such as AD, schizophrenia and should study other networks, such as the executive control network and the salience network, in addition to the DMN.

## Conclusion

We constructed the structural network of the DMN in this study and analyzed the relationship between the participants' MQ, SAS score, and the DMN. This study provides evidence for a significant role of the DMN in memory and anxiety, offering a perspective at the network level that showed that a higher MQ corresponded to higher global and local efficiencies of the DMN network, while a higher level of anxiety corresponded to lower global and local efficiencies.

### Conflict of interest statement

The authors declare that the research was conducted in the absence of any commercial or financial relationships that could be construed as a potential conflict of interest.
