# Supplementary material for: The Structural Connectivity Pattern of the Default Mode Network and Its Association with Memory and Anxiety
Source: Front Neuroanat. 2015 Nov 26;9:152. doi: 10.3389/fnana.2015.00152 (PMC4659898; doi:10.3389/fnana.2015.00152)
Supplement: Supplementary file 1 [file DataSheet1.DOCX]

***Supplementary Material***

**The structural connectivity pattern of the default mode network and its association with memory and anxiety**

**Yan Tao^1,2^, Bing Liu^1,2,*^, Xiaolong Zhang^1,2^, Jin Li^1,2^,Wen Qin^3^, Chunshui Yu^3^, Tianzi Jiang^1, 2,4, 5^**

^1^ Brainnetome Center, ^2^ National Laboratory of Pattern Recognition, Institute of Automation, Chinese Academy of Sciences, Beijing 100190, China

^3^ Department of Radiology, Tianjin Medical University General Hospital, Tianjin 300052, China

^4^ Queensland Brain Institute, The University of Queensland, Brisbane, QLD 4072, Australia

^5^ Key Laboratory for NeuroInformation of Ministry of Education, School of Life Science and Technology, University of Electronic Science and Technology of China, Chengdu 610054, China

^*^**Correspondence:** Bing Liu, Brainnetome Center, National Laboratory of Pattern Recognition,

Institute of Automation, Chinese Academy of Sciences, Beijing 100190, China

Phone: +86 10 8254 4770; Fax: +86 10 8254 4777

Email: [bliu@nlpr.ia.ac.cn](mailto:bliu@nlpr.ia.ac.cn)

1. **Supplementary Figures and Tables**

## Supplementary Tables

**Supplementary Table 1. Partial correlations between the MQ and some other properties under the threshold of 0.002 (*p* < 0.05, without correction for multiple comparisons)**

|  |  |  | ***pc*** | ***p-value*** |
| --- | --- | --- | --- | --- |
| **MQ** | **Average degree of node** | mPFC | 0.147 | 0.020 |
|  |  | PCC | 0.147 | 0.020 |
|  |  | right TP | 0.131 | 0.033 |
|  | **nodal efficiency** | mPFC | 0.159 | 0.013^*^ |
|  |  | PCC | 0.156 | 0.014^*^ |
|  |  | left TP | 0.121 | 0.045 |
|  |  | right TP | 0.175 | 0.007^*^ |
|  | **Structural connectivity** | mPFC-right suPF | 0.129 | 0.033 |
|  |  | right HIP-right TP | -0.192 | 0.002 |
|  |  | PCC-right TP | 0.188 | 0.004 |

*pc* refers to partial correlation coefficient. * means the correlation is significant under the false-discovery rate (FDR), q<0.05 threshold for correction of multiple comparisons

**Supplementary Table 2. Partial correlations between the SAS score and some other properties under the threshold of 0.002 (p < 0.05, without correction for multiple comparisons)**

|  | | | ***pc*** | ***p-value*** |
| --- | --- | --- | --- | --- |
| **SAS score** | **Average degree of node** | mPFC | -0.192 | 0.004^*^ |
|  |  | left supF | -0.125 | 0.040 |
|  |  | right supF | -0.127 | 0.038 |
|  |  | left HIP | -0.135 | 0.029 |
|  |  | left TP | -0.129 | 0.036 |
|  |  | right TP | -0.140 | 0.025 |
|  | **nodal efficiency** | mPFC | -0.190 | 0.004^*^ |
|  |  | left supF | -0.123 | 0.043 |
|  |  | right supF | -0.125 | 0.040 |
|  |  | left HIP | -0.138 | 0.027 |
|  |  | right TP | -0.120 | 0.047 |
|  | **Structural connectivity** | mPFC-left supF | -0.120 | 0.045 |
|  |  | right supF-left HIP | -0.125 | 0.038 |
|  |  | right supF-right TP | -0.119 | 0.046 |
|  |  | right HIP-left TP | -0.117 | 0.049 |

*pc* refers to partial correlation coefficient. * means the correlation is significant under the false-discovery rate (FDR) threshold for correction of multiple comparisons
